# Significance of Hyponatremia as an Independent Factor in Predicting Short-term Mortality in Patients with Hemorrhagic Stroke

**DOI:** 10.7759/cureus.4549

**Published:** 2019-04-27

**Authors:** Ali Shah, Samurna Sabir, Moiz Artani, Osama Salam, Shehroz Khan, Amber Rizwan

**Affiliations:** 1 Internal Medicine, Jinnah Sindh Medical University, Karachi, PAK; 2 Internal Medicine, Dow University of Health Sciences, Karachi, PAK; 3 Community Health Sciences, Jinnah Medical and Dental College, Karachi, PAK; 4 Family Medicine, Civil Hospital, Karachi, PAK

**Keywords:** hyponatremia, mortality risk, in-hospital mortality, hemorrhagic stroke, predictors of mortality, disease outcome, stroke, prognosis, intracerebral hemorrhage

## Abstract

Introduction

Hyponatremia is the most common electrolyte imbalance in patients with acute cerebrovascular insults. In patients with acute non-traumatic hemorrhagic stroke, the role of hyponatremia as a negative prognostic indicator has been controversial. The aim of this study is to evaluate the frequency of hyponatremia in patients with hemorrhagic stroke and determine its impact on their in-hospital disease outcome.

Methods

This was a prospective observational study, which included all patients with non-traumatic hemorrhagic stroke. Serum sodium concentration <135 mmol/L was labeled as hyponatremia. The etiology of hyponatremia was determined as syndrome of inappropriate antidiuretic hormone (SIADH) and cerebral salt wasting syndrome (CSWS). The outcome was categorized as “complete recovery,” “motor/sensory deficit but not wheelchair/bed bound,” “wheelchair/bed bound,” and “in-hospital mortality.” SPSS for Windows version 22.0 (IBM Corp., Armonk, NY, US) was used to analyze the data.

Results

Out of 234 patients of hemorrhagic stroke, 45.3% (n=106) were hyponatremic, 58.5% had SIADH and 41.5% had CSWS. The overall mortality rate of hemorrhagic stroke was 16.2%. The mortality rate was 36.5% in the SIADH group, 50% in the CSWS group, and 13.1% in the normonatremic group (p<0.00001). The mean hospital stay in the SIADH group was 7.04 ± 2.57, in the CSWS group, it was 6.50 ± 1.55, and in the normonatremic group, it was 3.88 ± 2.74 (p=0.000).

Conclusion

Hyponatremia is an independent predictor of short-term mortality in patients with acute hemorrhagic stroke.

## Introduction

Hyponatremia (serum sodium <135mmol/l) is the most common electrolyte imbalance encountered not only in the general population but also in patients with acute ischemic and hemorrhagic stroke. Hyponatremia may be due to syndrome of inappropriate antidiuretic hormone (SIADH) or cerebral salt wasting syndrome (CSWS) [1-2].

Antidiuretic hormone (ADH) is a hormone of the pituitary gland that regulates water quantities in the body by acting on the kidneys. Physiologically, ADH is secreted in response to decreased plasma volume or increased serum osmolality. In SIADH, ADH is persistently secreted due to a failure of the negative feedback mechanism leading to increased water resorption from the kidneys and, consequently, dilutional hyponatremia [3]. On the other hand, hyponatremia in CSWS is due to large urinary losses of sodium [1].

Hyponatremia has been described as one of the risk factors predisposing to cerebrovascular stroke. Hyponatremia is also a complication of stroke. It has a crucial impact on the prognosis as well as the short-term and long-term mortality in patients with stroke [4]. In patients with hemorrhagic stroke (intracerebral hemorrhage (ICH)), hyponatremia was first evaluated by Kuramatsu et al. in 2014 [5]. They reported that in-hospital mortality was twice as common in hyponatremic patients as compared to normonatremic patients and the hyponatremia correction does not influence mortality [5]. This was a breakthrough publication which was succeeded by a few other research works, which reported higher mortality in hyponatremic patients of ICH [6-7].

Clinical data is still scarce. To the best of our knowledge, there exists only one study from Pakistan that evaluated the serum sodium levels in patients with ischemic stroke and found that 25% were mildly, 9.8% were moderately, and 3.8% were severely hyponatremic. They did not correlate serum sodium levels with either duration of hospital stay or short- or long-term mortality [8]. However, we could not come across any study predicting the association of hyponatremia with mortality in patients with ICH. The aim of this study is to evaluate the frequency of hyponatremia in patients with hemorrhagic stroke and determine their short-term outcome.

## Materials and methods

This was a prospective observational study, which was conducted in the department of medicine in a tertiary care public hospital in Karachi over a period of six months (July-Dec 2018). The study was approved by the institutional review board, which waived patient consent. During the study period, there were 638 patients admitted with a clinical and neuro-radiological diagnosis of stroke. Upon neuroimaging, there were 234 (36.6%) established cases of hemorrhagic stroke that were included in the study and evaluated for hyponatremia. Serum sodium concentration <135 mmol/L was labeled as hyponatremia [1]. Patients with a history of gastroenteritis, head trauma, brain tumor, pulmonary Kochs, bacterial pneumonia, bronchogenic carcinoma, leukemia, lymphoma, recent surgery, and patients who were taking any drugs that can cause hyponatremia were excluded from this study. Patients who died within the hospital emergency room and patients who died so early after admissions that their baselines labs weren’t sent to or chased from the laboratory were also excluded, as even one serum sodium value was not available.

Hence, out of 234 cases of hemorrhagic stroke, 106 (45.3%) hyponatremic patients aligned with the inclusion criteria and were included. When the patients were categorized according to the etiology of hyponatremia, CSWS was identified in 44 (41.5%) patients, and SIADH was identified in 62 (58.5%) patients. Extracellular fluid volume, hematocrit, serum electrolytes, serum albumin, serum uric acid, and renal functions tests were included for all patients. CSWS and SIADH were evaluated as given in Table 1, which is adapted from Saleem et al. [1].

**Table 1:** Clinical parameters for the diagnosis of CSWS and SIADH BUN: Blood urea nitrogen; SIADH: Syndrome of Inappropriate secretion of antidiuretic hormone; CSWS: Cerebral salt wasting syndrome

Clinical Parameters	CSWS	SIADH
Hematocrit	Increased	Normal
Serum albumin concentration	Increased	Normal
BUN / creatinine	Increased	Decreased
Serum potassium	Normal or increased	Normal
Serum uric acid	Normal or increased	Decreased
Treatment	Normal saline	Fluid restriction

The site of hemorrhage was determined on magnetic resonance imaging (MRI) or magnetic resonance angiography (MRA). Non-contrast computed tomography (NCCT) was done for patients who were unable to tolerate MRI due to claustrophobia or had contraindications to it, including aneurysm clips, pacemakers, rods and screws, or any other magnetic materials in their bodies, or were on ventilatory life support as NCCT becomes more practical in these patients. Hemorrhagic sites identified for this study included left and right putamen, thalamic, and cerebellar hemorrhage and pontine hemorrhage. Patients were followed throughout their hospital stay to evaluate their duration of stay and outcome of stroke. The outcome was categorized as “complete recovery” when there was no deficit on examination; “motor/sensory deficit but not wheelchair/bed bound” when there was minor deficit upon discharge although the patient was not dependent; “wheelchair/bed bound” when the deficit was severe enough to render the patient wheelchair-bound or bedridden; and “in-hospital mortality” when the patient died after 24 hours of hospital admission. SPSS for Windows version 22.0 (IBM Corp., Armonk, NY, USA) was used to analyze the data. Frequencies and percentages were calculated for categorical variables and mean and standard deviation (SD) were calculated for continuous variables. Serum sodium status of the patients was compared to the hemorrhage site and in-hospital outcome to deduce any relationships. Statistical significance for these relationships was calculated using Chi-square. P-value of ≤0.05 was taken as significant.

## Results

The incidence of hyponatremia in patients with hemorrhagic stroke was 45.3% (n=106). Out of these 106 patients, there were 83 (78.3%) men and 23 (21.7%) women. The male to female ratio was 3.5:1. Their mean age was 59.14 ± 9.05 years with men being slightly older (mean age: 60.55 ± 4.81 years) than women (mean age: 59.07 ± 1.11 years). There were 13 (12.3%) patients who have had a previous episode of stroke.

Patients were then categorized according to their site of hemorrhage. Overall, left putamen hemorrhage was the most common (n=56; 52.8%) and pontine hemorrhage was the least common (n=8; 7.5%). Among the patients with hyponatremia due to SIADH, right putamen hemorrhage was the most common (n=14; 22.5%), followed by left putamen (n=13; 20.9%) and left thalamic hemorrhage (n=11; 17.7%). Among the patients with hyponatremia due to SIADH, left putamen hemorrhage was the most common (n=11; 25%), followed by right putamen (n=9; 20.4%) and right thalamic hemorrhage (n=8; 18.2%). Left putamen hemorrhage was also the most common site in patients who did not develop hyponatremia (n=32; 25%) followed by right thalamic hemorrhage (n=27; 21%). There was no statistical significance in the distribution of the hemorrhage site as compared to the status of serum sodium as shown in Table 2.

**Table 2:** Distribution of hemorrhagic site according to the frequency of hyponatremia due to SIADH and CSWS and patients with no hyponatremia SIADH: Syndrome of Inappropriate secretion of antidiuretic hormone; CSWS: Cerebral salt wasting syndrome

Site of hemorrhagic stroke	Hyponatremia due to SIADH (n=62)	Hyponatremia due to CSWS (n=44)	Patients with no hyponatremia (n=128)	P value
Right putamen hemorrhage	14 (22.5%)	9 (20.4%)	23 (17.9%)	0.74
Left putamen hemorrhage	13 (20.9%)	11 (25%)	32 (25%)	0.81
Right thalamic hemorrhage	10 (16.2%)	8 (18.2%)	27 (21.0%)	0.70
Left thalamic hemorrhage	11 (17.7%)	7 (15.9%)	14 (10.9%)	0.39
Right cerebellar hemorrhage	9 (14.5%)	5 (11.3%)	16 (12.5%)	0.88
Left cerebellar hemorrhage	3 (4.8%)	1 (2.2%)	13 (10.2%)	0.15
Pontine hemorrhage	2 (3.2%)	3 (6.8%)	3 (2.3%)	0.36

The outcome of stroke was categorized on four levels and compared to the serum sodium status. The mortality rate of hemorrhagic stroke was 16.2%; 50% of the deceased patients had hyponatremia due to CSWS. There were 20.9% who were rendered wheelchair or bed-bound; 38.7% of these did not develop hyponatremia. There were 42.7% of patients who had a motor or sensory deficit but not wheelchair or bed bound; 73% of these did not develop hyponatremia; and 20% had hyponatremia due to SIADH. Only 20.1% of the patients recovered completely; 66% of these did not develop hyponatremia, 27.6% had hyponatremia due to SIADH, and 6.3% had hyponatremia due to CSWS. The comparisons of the mean duration of hospital stay and the outcomes of stroke among the three groups of patients are shown in Table 3.

**Table 3:** Outcome of stroke in patients with hyponatremia due to SIADH and CSWS and patients with no hyponatremia SIADH: Syndrome of Inappropriate secretion of antidiuretic hormone; CSWS: Cerebral salt wasting syndrome

Outcome of Stroke (N=234)	Hyponatremia due to SIADH	Hyponatremia due to CSWS	Patients with no hyponatremia	P value
Mean duration of hospital stay in days	7.04 ± 2.57	6.50 ± 1.55	3.88 ± 2.74	0.000
Complete recovery (n=47; 20.1%)	13 (27.6%)	3 (6.3%)	31 (66%)	0.04
Motor / sensory deficit but not wheelchair/bed bound (n=100; 42.7%)	20 (20%)	7 (7%)	73 (73%)	<0.00001
Wheelchair/bed bound (n=49; 20.9%)	15 (30.6%)	15 (30.6%)	19 (38.7%)	0.01
In-hospital mortality (n=38; 16.2%)	14 (36.8%)	19 (50%)	5 (13.1%)	<0.00001

## Discussion

One of the most common acute electrolyte imbalances in patients with stroke is hyponatremia. It mimics the signs of underlying neurological deficit and worsens the disease outcome [1-2,5]. This study evaluated that almost half of the patients with hemorrhagic stroke developed hyponatremia. SIADH was a more common entity. The duration of hospital stay was significantly longer in patients with hyponatremia. Not only was the mortality rate higher in these patients, but the outcome of surviving patients, at the time of discharge, was worse in patients with hyponatremia.

This study is crucial is establishing hyponatremia as a negative prognostic factor in patients with non-traumatic hemorrhagic stroke. However, it has its limitations. The study followed the patients till discharge and has only reported short-term outcomes in these patients. It was a single-center, time-bound study, which was limited by the number of patients available for analysis. To establish hyponatremia as a negative prognostic factor and predictor of mortality, patients should have been followed for a longer term. Furthermore, not all patients had central venous pressures (CVP) measured, hence, the diagnosis of SIADH vs. CSWS might not be absolute. When CVP was not available, we used the fluid balance to distinguish SIADH from CSWS. Hence, our results are more clinical and based on real-world practices.

Hyponatremia was a previously unrecognized predictor of hospital stay and in-hospital mortality in patients with hemorrhagic stroke and was first studied in 2014 [5]. Since then, hyponatremia has been gaining wide recognition as a negative prognosis in cerebrovascular accidents. The frequency of hyponatremia has been reported from 12%-52% of patients with ischemic or hemorrhagic stroke. The frequency of hyponatremia in this is comparable to the literature [1-2,4-9]. We had a higher incidence of SIADH (58.5%) as compared to CSWS (41.5%).

The first time hyponatremia was established as a predictor of mortality in patients with hemorrhagic stroke, these patients were seen to have twice the mortality rate as compared to normonatremic patients [5]. The second study done to reinforce this association reported a higher mortality rate in normonatremic patients as compared to hyponatremic (32% vs. 24%), the difference was not statistically significant. However, hyponatremic patients had a significantly longer median duration of hospital stay [7]. Carcel et al. then studied the effect of hyponatremia in the outcome of hemorrhagic stroke in a large sample. At 90 days, the mortality rate in the hyponatremic group was 18% and in the normonatremic group was 11%. Hyponatremic patients had a 1.8 times higher risk of death as compared to the normonatremic patients (p<0.001). However, their hyponatremic patients did not have a statistically significant risk of major disability after multivariable adjustment [6]. However, Kalita et al. failed to show hyponatremia as a negative prognostic factor of stroke. In their study, death was higher in hypernatremic patients as compared to hyponatremic and normonatremic patients (p=.04) [9].

Whether or not hyponatremia can be established as a negative indicator of short- and long-term prognosis of hemorrhagic stroke is still not established through literature. Some literature does report otherwise; however, most studies have proposed the association. This literature helps neurologists and medical specialists to identify high-risk patients earlier, at the time of admission. Periodic serum sodium monitoring and in-time interventions can help prevent adverse outcomes in these patients.

## Conclusions

Hyponatremia is a very common electrolyte imbalance reported in patients with acute cerebrovascular insult. The clinical significance of hyponatremia was undermined previously. However, current research has helped in establishing the crucial role of serum sodium levels in predicting short-term outcomes in patients with acute hemorrhagic stroke. Close monitoring of serum sodium levels in these patients, determination of the etiology of hyponatremia, and appropriate medical intervention can improve the outcome in these patients.
